# Differential modulation of cytokine, chemokine and Toll like receptor expression in chickens infected with classical and variant infectious bursal disease virus

**DOI:** 10.1186/1297-9716-42-85

**Published:** 2011-07-12

**Authors:** Abdul Rauf, Mahesh Khatri, Maria V Murgia, Kwonil Jung, Yehia M Saif

**Affiliations:** 1 Food Animal Health Research Program, Ohio Agricultural Research and Development Center, The Ohio State University, 1680 Madison Avenue, Wooster, OH 44691, USA; 2 Department of Veterinary Preventive Medicine, College of Veterinary Medicine, The Ohio State University, Columbus, OH 43210, USA

## Abstract

Infectious bursal disease (IBD) is an important immunosuppressive disease of chickens. The causative agent, infectious bursal disease virus (IBDV), consists of two serotypes, 1 and 2. Serotype 1 consists of classic IBDV (cIBDV) and variant IBDV (vIBDV). Both of these strains vary in antigenicity and pathogenesis. The goal of this study was to compare the immunopathogenesis of cIBDV and vIBDV. Three-week-old specific pathogen free chickens were inoculated intraocularly with standard challenge strain (STC) (cIBDV) and a variant strain Indiana (IN) (vIBDV). The cIBDV produced more pronounced bursal damage, inflammatory response and infiltration of T cells as compared to vIBDV. There were significant differences in the expression of innate (IFN-α and IFN-β), proinflammatory cytokine and mediator (IL-6 and iNOS) in cIBDV- and vIBDV-infected bursas. The expression of chemokines genes, IL-8 and MIP-α was also higher in cIBDV-infected chickens during the early phase of infection. The expression of Toll like receptor 3 (TLR3) was downregulated at post inoculation days (PIDs) 3, 5, and 7 in the bursas of vIBDV-infected chickens whereas TLR3 was upregulated at PIDs 3 and 5 in cIBDV-infected bursas. In vIBDV-infected bursa, TLR7 expression was downregulated at PIDs 3 and 5 and upregulated at PID 7. However, TLR7 was upregulated at PIDs 3 and 7 in cIBDV-infected bursas. The expression of MyD88 was downregulated whereas TRIF gene expression was upregulated in cIBDV- and vIBDV-infected bursa. These findings demonstrate the critical differences in bursal lesions, infiltration of T cells, expression of cytokines, chemokines and TLRs in the bursa of cIBDV-and vIBDV-infected chickens.

## Introduction

Infectious bursal disease (IBD) is one of the most important naturally occurring viral diseases of commercial chickens worldwide [1]. The causative agent, IBD virus (IBDV) belongs to the family *Birnaviridae*. The virus causes an acute, highly contagious and immunosuppressive disease in chickens [1]. The virus infects and destroys actively dividing IgM-bearing B cells in the bursa of fabricius [2,3]. IBDV exists in different antigenic and pathogenic forms [4]. Initial isolates designated as classical strains (cIBDV) of the serotype 1 viruses were considered to be a single antigenic type. In the early 1980s, antigenic variants (vIBDV) of the virus were identified in the United States [1]. These variant viruses were able to cause disease in the presence of immunity to cIBDV viruses [5]. The antigenic variants typically do not cause clinical signs of disease but can cause a marked immunosuppression [4]. The immunosuppression caused by variants and classical strains of IBDV is often associated with secondary viral infections and bacterial infections [6-11]. The IBDV induced immunosuppression also renders chicken flocks refractory to live attenuated vaccines against other viral diseases such as avian influenza virus, infectious bronchitis and Newcastle disease virus [12,13].

Following infection and replication of IBDV, T cells infiltrate the bursa of infected chickens [14,15]. Although B cells are considered the major targets for IBDV, it has been shown that the virus can infect and possibly replicate in macrophages [16-18]. Following viral infections, including IBDV, activated macrophages produce various mediators, such as proinflammatory cytokines, interleukin-1 (IL-1) and IL-6, chemokines, and nitric oxide (NO) [16,19-21].

Host cells use various receptors to detect viral infections by recognizing pathogen-associated molecular patterns (PAMPs) and subsequently induce an antiviral response. Prominent among these are Toll-like receptors (TLRs) [22-24]. Several TLRs recognize viral PAMPs: TLR3, detects double-stranded RNA (dsRNA) derived from viral replication whereas single-stranded RNA (ssRNA) are detected by TLR7 and TLR8 [22]. The TLR signaling proceeds via two pathways; the myeloid differentiation factor 88 (MyD88)-mediated pathway and the Toll-interleukin-1 receptor (TIR)-domain-containing adaptor inducing IFN-*β *(TRIF)-mediated pathway [25,26]. The TLR signaling pathways arise from intracytoplasmic TIR domains, which are conserved among all TLRs. The TLR7 specifically involves MyD88-dependent pathway, whereas TRIF is implicated in the TLR3-mediated MyD88-independent pathway [27].

The IBD is controlled by vaccination and the vaccines are very effective against classical strains but with the emergence of variant and the very virulent strains of IBDV in the United States [28], there are several incidents of vaccine failure [28-32]. This highlights the need to examine the differential immuno-pathogenesis of classical and variant strains of IBDV in order to devise better control strategies. Limited information is available on the comparative pathogenesis of cIBDV and vIBDV. Sharma et al. [33] reported that similar to cIBDV, vIBDV also suppressed the ability of T cells to respond to mitogens and the bursal lesions induced by cIBDV were accompanied by infiltration of inflammatory cells whereas inflammatory cells infiltration was lacking in the bursa of vIBDV-infected chickens [33].

In this study, we examined the differential immuno-pathogenesis of classical and variant strains of IBDV. As compared to vIBDV, cIBDV induced early bursal lesions, extensive infiltration of T cells in the bursa and induced higher expression of proinflammatory cytokine and mediators; IL-6 and iNOS. Further, there were differences in the expression of TLR3 and TLR7 and their adapter molecules, TRIF and MyD88, in the bursa of cIBDV and vIBDV-infected chickens. These data demonstrate the differential induction of innate and T cell responses by cIBDV and vIBDV. Elucidation of the TLRs signaling pathway and factors leading to activation of the immune response to IBDV infection may provide new strategies for the development of cross-protective vaccines that can augment T cell responses in addition to an antibody response.

## Materials and methods

All protocols of this study were designed and performed in accordance with animal use protocol number 08-Ag-029, approved by the Agricultural Animal Care and Use committee, The Ohio State University.

### Chickens and virus strains

Specific pathogen free (SPF) chicken eggs (Charles River Laboratories Inc; Wilmington, MA, USA) were incubated and hatched in our facility at The Ohio Agricultural Research and Development Center, The Ohio State University. Chickens were kept in a disease containment building. At 3-weeks of age, prior to inoculation with virus, the chickens were transferred to an isolation unit. The standard challenge strain, (STC) [34] representing cIBDV strain, and a variant Indiana (IN) representing (vIBDV), were propagated in chickens and titrated in eggs as described earlier [32].

### Experimental design

Eighty-four SPF chickens were allocated to 3 groups; 36 chickens in both group 1 and group 2 were inoculated intraocularly with 10^4 ^EID_50_/200 uL of either vIBDV or cIBDV strains respectively and 12 chickens in group 3 were inoculated similarly with PBS to serve as virus- free controls [35]. At post inoculation days (PIDs) 3, 5 and 7, twelve chickens each from the virus-infected groups and four chickens from the virus-free group were euthanized and bursas were collected. Four pools of three bursas each from virus-infected groups were prepared. The harvested bursal tissues were examined for the following: 1- histopathological lesions, 2- immunohistochemical detection of virus antigen, T cells and macrophages, 3- isolation of mononuclear cells and expression of virus-induced innate, proinflammatory cytokines, chemokines, Toll like receptors (TLRs), and their adaptor molecules by quantitative real time RT-PCR (qRT-PCR).

### Microscopic lesions

At PIDs 3, 5 and 7, four bursas each from virus-free control, cIBDV- and vIBDV-infected chickens were harvested, fixed in 10% phosphate buffered formalin and stained with hematoxylin and eosin for the detection of histopathological lesions. Bursal follicular lesions were observed microscopically and lesion scores were determined. The bursal lesions were scored as follows: lesion score 1 represents 1-25% of lymphoid follicles affected, 2 represents 26-50% of lymphoid follicles affected, 3 represents 51-75% of lymphoid follicles affected and 4 represents 76-100% of lymphoid follicles affected [36,37].

### Detection of IBDV-antigen, macrophages and T cells in virus-infected bursas

The IBDV antigens, T cells, and macrophages were detected in snap frozen sections of bursas of vIBDV-, cIBDV-infected and virus-free control chickens by immunohistochemistry [16,38]. Briefly, small sections of bursal tissue were embedded in Tissue-Tek^® ^O.C.T compound (Sakura Finetek, CA, USA), sectioned 5 μM thick with the help of cryostat microtome (Leica CM 1510S Germany), spotted on double positive glass slides, and dehydrated at 37°C overnight. The dehydrated sections were fixed with acetone (75% acetone and 25% ethyl alcohol) for 10 min followed by 3 washes with phosphate buffered saline (PBS). The tissue sections were then blocked with 2% goat serum for 1 h. After blocking, the sections were incubated with respective primary and secondary antibodies. A biotin-streptavidin-peroxidase method using R.T.U vectastain^(R) ^kit (Vector Laboratories, Burlingame, CA, USA) was adopted for the detection of viral antigen, T cells and macrophages in frozen sections of vIBDV-, cIBDV-infected and virus-free chickens bursas. The primary antibodies used for the detection of T cells and macrophages were: mouse anti-chicken CD3, (diluted 1:200) and mouse anti-chicken monocytes/macrophage KUL01, (diluted 1:400) (Southern Biotech, Birmingham, AL, USA). The primary antibody used for the detection of IBDV antigen was biotinylated mouse anti-IBDV polyclonal antibody raised against IN strain of IBDV which reacts with both cIBDV and vIBDV (diluted 1:100). The development of dark brown color indicated a positive reaction. The group mean ± SEM of vIBDV- and cIBDV-infected cells, macrophage or T cells per field was determined at 20 × magnification after counting 5 fields/bursa/chicken and compared with virus-free control groups.

### Isolation of bursal mononuclear cells

Twelve bursas either from cIBDV- or vIBDV-infected groups at each PID were pooled into 4 pools of three bursas each and bursas were also collected from 4 virus-free control chickens at each PID. Mononuclear cells were isolated from bursas as previously described [16,38]. Briefly, mononuclear cells suspension was prepared from bursas by density gradient centrifugation (gradient density 1.090) over Ficoll-Hypaque (GE healthcare Bio-Sciences, Uppsala, Sweden) and washed twice in cold RPMI 1640 (Gibco, Carlsbad, CA, USA). The cell pellets were lysed with Trizol reagent and stored at -70 for RNA extraction.

### RNA extraction and qRT-PCR

Total RNA from bursal mononuclear cells of virus-infected and virus-free control chickens was extracted using the Trizol reagent (Invitrogen, Carlsbad, CA, USA) according to the manufacturer's instructions. qRT-PCR was used for the quantification of genes specific for the expression of messenger RNAs (mRNAs) for innate (IFN-α, IFN-β) and proinflammatory (IL-6 and iNOS) cytokines, chemokines (IL-8 and MIP-α), Toll like receptors and their adaptor molecules (TLR3, TLR7, MyD88 and TRIF) [16]. The primers for 28S, IFN-α, IFN-β, IL-6, iNOS, IL-8, MIP-α, TLRL3, TLR7, TRIF and MyD88 were designed according to previously published sequences [39-41]. RT-PCR was performed using Power SYBER^® ^Green RNA - to-C_T _™ 1 step RT-PCR kit (Applied Biosystems, Foster City, CA, USA). Amplification and detection were performed in an automated 7500 Real time RT-PCR system (Applied Bio System, Foster City, CA, USA). Fold increase of target gene expression over uninfected controls was calculated with the 2^-^ΔΔC_T _method [16,17,38,42].

### Statistical analysis

Graph Pad Prism version 5 for Windows was used for graphical presentation of data. Student's *t*-test was used to detect significant differences between vIBDV- and cIBDV-infected chickens. *P *< 0.05 was considered to be statistically significant.

## Results

### Infection of chickens with cIBDV and vIBDV

**Figure 1:**
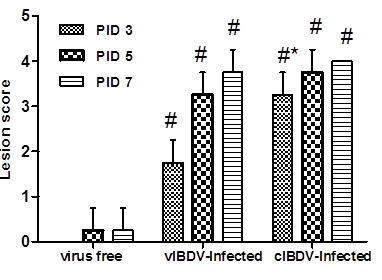
**Histopathological lesions in the bursa following IBDV-infection**. Bursal lesions score of follicular depletion in virus-free control, vIBDV and cIBDV-infected chickens at PIDs 3, 5 and 7. ^#^Statistically significant differences between control and vIBDV- or cIBDV-infected groups (*p *< 0.05). *Statistically significant differences between vIBDV- and cIBDV-infected groups (*p *< 0.05).

Inoculation of chickens with vIBDV and cIBDV resulted in a typical IBDV infection. All of the chickens inoculated with cIBDV showed morbidity and 8% mortality whereas chickens infected with vIBDV appeared healthy and no morbidity or mortality was noted in this group. Virus-free control chickens had no clinical signs or macroscopical lesions during the course of the experiment. Histologically, cIBDV induced early and more pronounced bursal damage as compared to vIBDV (Figure 1). Severe follicular lesions were observed histologically in the bursa of cIBDV-infected chickens at PID 3 with a mean bursal lesions score of 3.25 ± 0.50 whereas in vIBDV-infected group, the lesion score at PID 3 was 1.75 ± 0.50. At PIDs 5 and 7, the lesion score increased to 3.75 ± 0.50 and 4 ± 00 respectively in cIBDV-infected bursa. In vIBDV-infected bursa, lesion score was 3.25 ± 0.50 and 3.75 ± 0.50 at PIDs 5 and 7, respectively (Figure 1).

### Detection of IBDV-antigen, T cells and macrophages in virus-infected bursa

**Figure 2:**
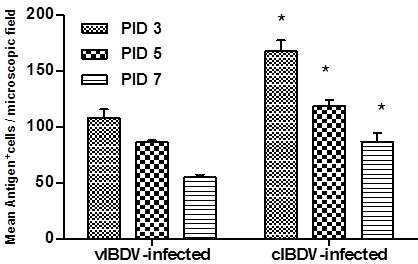
**IBDV antigen detection by immunohistochemical staining in virus-infected bursas**. SPF chickens were inoculated with 10^4^EID_50 _of cIBDV or vIBDV and bursal tissues were collected at PIDs 3, 5 and 7. Bursal sections from virus-free chickens, vIBDV- and cIBDV-infected chickens were examined for the presence of IBDV antigen by immunohistochemistry. IBDV positive cells were counted (40X) at PID 3, 5 and 7. The values represent the mean ± SEM of 5 fields/bursa/chicken on designated PID. *Statistically significant differences between vIBDV- and cIBDV-infected groups (*p *< 0.05).

**Figure 3:**
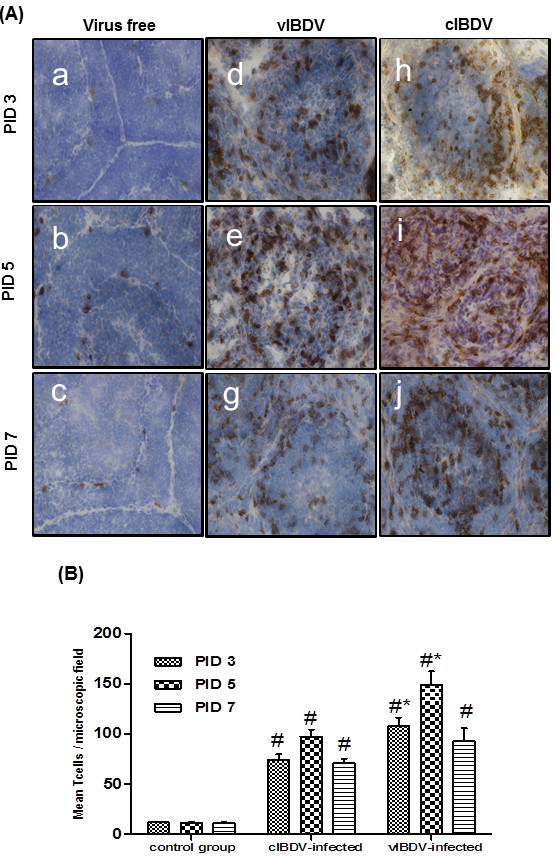
**T cell infiltration in IBDV-infected bursa**. SPF chickens were inoculated with 10^4^EID_50 _of cIBDV or vIBDV and bursal tissues were collected at PID 3, 5 and 7. (A) Bursal sections from virus-free chickens (**a, b **and **c; **20X), vIBDV-infected bursa (**d, e**, and **f; **20X) and cIBDV-infected bursa (**g, h**, and **i; **20X) were examined for the presence of T cells by immunohistochemistry using anti-chicken CD3^+ ^monoclonal antibody. Brown color indicates the positive staining. (B) T cells were counted (40X) at PID 3, 5 and 7. The values represent the mean ± SEM of 5 fields/bursa/chicken on designated PID. ^#^Statistically significant differences between control and vIBDV- or cIBDV-infected groups (*p *< 0.05). *Statistically significant differences between vIBDV- and cIBDV-infected groups (*p *< 0.05).

**Figure 4:**
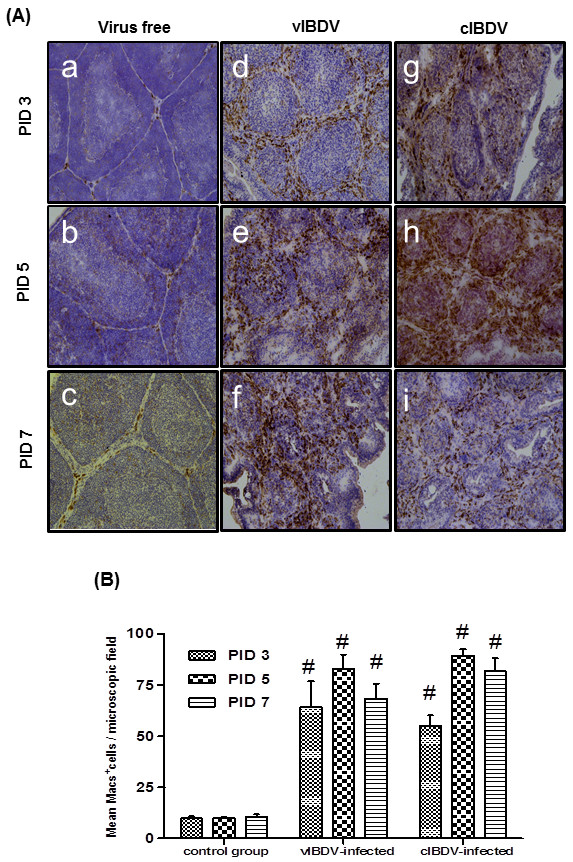
**Infiltration of macrophages in IBDV-infected bursa**. SPF chickens were inoculated with 10^4^EID_50 _of cIBDV or vIBDV and bursal tissues were collected at PID 3, 5 and 7. (A) Bursal sections from virus-free chickens (**a, b **and **c; **20X), vIBDV-infected chickens (**d, e**, and **f; **20X) and cIBDV-infected chickens (**g, h**, and **i; **20X) were examined for the presence of macrophages by immunohistochemistry using anti-chicken macrophage antibody. Brown color indicated by arrow represents the positive staining. (B) Macrophage positive cells were counted (20X) at PIDs 3, 5 and 7. The values represent the mean ± SEM of 5 fields/bursa/chicken on designated PID. ^#^Statistically significant differences between control and vIBDV- or cIBDV-infected groups (*p *< 0.05). *Statistically significant differences between vIBDV- and cIBDV-infected groups (*p *< 0.05).

We detected viral antigen, T cells and macrophages in cIBDV- and vIBDV-infected bursa at PID 3, 5 and 7 by immunohistochemistry. Number of IBDV antigen positive cells was significantly higher (*P *< 0.05) in cIBDV-infected bursa as compared to bursa of vIBDV-infected chickens at all the PIDs tested (Figure 2). Similar to viral antigen positive cells, we observed significantly higher (*P *< 0.05) number of infiltrating T cells in the bursa of cIBDV-infected chickens as compared to vIBDV-infected chickens at PID 3 and 5 (Figures 3A and 3B). However, both viral strains induced infiltration of similar number of macrophages in the bursa which was maximum at PID 5 (Figures 4A and 4B).

### Virus induced expression of innate cytokines

**Figure 5:**
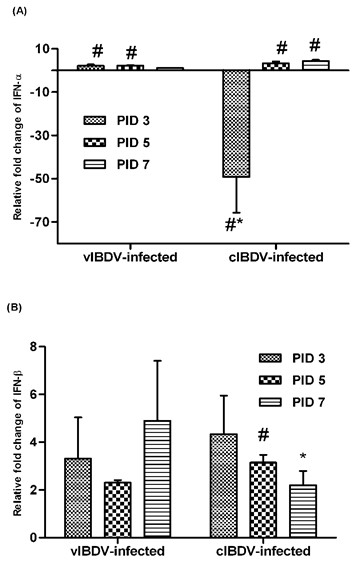
**Relative gene expression at mRNA level, of innate cytokines mRNA in IBDV-infected bursa**. At PIDs 3, 5 and 7, bursal mononuclear cells were isolated from cIBDV- or vIBDV-infected and virus-free control chickens and examined for (A) IFN-α and (B) IFN-β gene expression by qRT-PCR. Results are shown as transcription of the target gene relative to housekeeping gene 28S. The data are expressed as fold change expression in infected chickens over virus-free control. The values represent the mean ± SEM of 4 pools of 3 bursa each at designated PID. ^#^Statistically significant differences between control and vIBDV- or cIBDV-infected groups (*p *< 0.05). *Statistically significant differences between vIBDV- and cIBDV-infected groups (*p *< 0.05).

Both vIBDV and cIBDV induced innate cytokine response in virus infected chicken bursas. The innate cytokine IFN-α was upregulated at PID 3, 5 and 7 in the bursa of vIBDV-infected chickens (Figure 5A). In cIBDV-infected chickens, IFN-α was downregulated at PID 3 and upregulated at PID 5 and 7 (Figure 5A). IFN-β was upregulated in cIBDV- and vIBDV-infected chickens at PIDs 3, 5 and 7 (Figure 5B).

### Expression of proinflammatory cytokine, mediator and chemokines in IBDV-infected bursa

**Figure 6:**
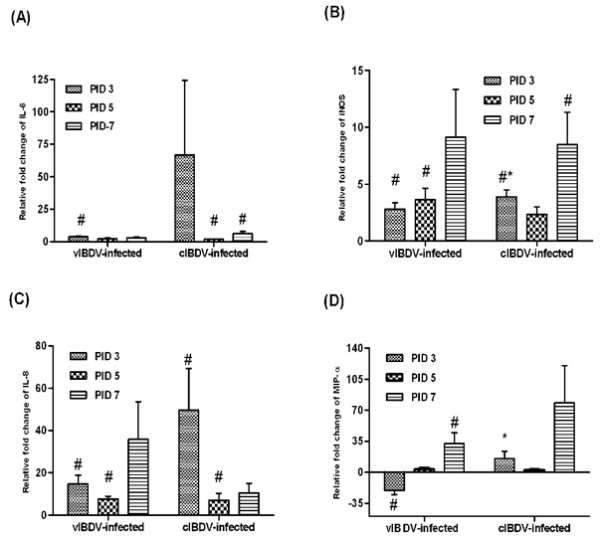
**Relative gene expression at mRNA level, of proinflamatory cytokine, iNOS, and chemokine mRNA in IBDV-infected bursa**. At PIDs 3, 5 and 7, bursal mononuclear cells were isolated from cIBDV, vIBDV and virus-free control chickens and examined for IL-6 (A), iNOS (B), IL-8 (C), and MIP-α (D) gene expression by qRT-PCR. Results are shown as transcription of the target gene relative to housekeeping gene 28S. The data are expressed as fold change expression in infected chickens over virus-free control. The values represent the mean ± SE of 4 pools of 3 bursa each at designated PID. ^#^Statistically significant differences between control and vIBDV- or cIBDV-infected groups (*p *< 0.05). *Statistically significant differences between vIBDV- and cIBDV-infected groups (*p *< 0.05).

Classical strains of IBDV are known to induce a strong inflammatory response in the bursa. In this study, we detected stronger upregulation in the gene expression of IL-6 in the bursa of cIBDV-infected chickens than vIBDV-infected chickens during the early stage of infection (Figure 6A). Similarly, cIBDV induced significantly higher (*P *< 0.05) expression of iNOS as compared to vIBDV at PID 3 (Figure 6B). Expression of the chemokine IL-8 was also higher in cIBDV-infected bursa at PID 3 (Figure 6C). MIP-α expression was significantly elevated (*P *< 0.05) in cIBDV-infected chickens at PID 3 and substantially elevated at PID 7 as compared to vIBDV-infected chickens (Figure 6D). These data suggest that both viral strains activate proinflammatory cytokine and chemokine responses which are more pronounced in cIBDV-infected chickens.

### Expression of toll like receptors and adaptor molecules in virus-infected bursa

**Figure 7:**
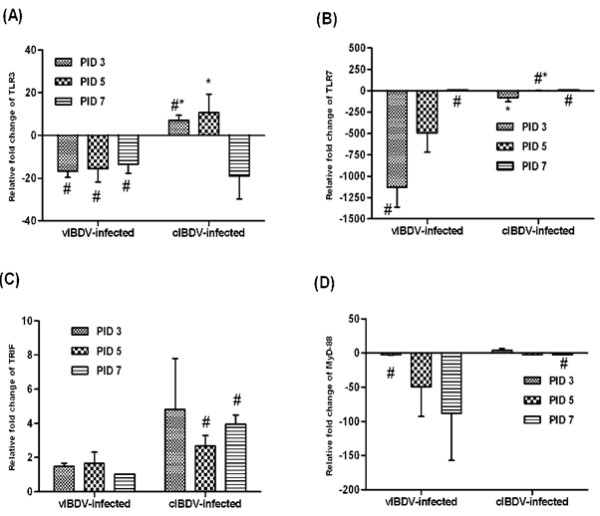
**Relative gene expression of Toll like receptor and adaptor molecule mRNA in IBDV-infected bursa**. At PIDs 3, 5 and 7, bursal mononuclear cells were isolated from cIBDV, vIBDV and virus-free control chickens and examined for TLR3 (A), TLR7 (B), TRIF (C), and MyD88 (D) gene expression by qRT-PCR. Results are shown as transcription of the target gene relative to housekeeping gene 28S. The data are expressed as fold change expression in infected chickens over virus-free control. The values represent the mean ± SE of 4 pools of 3 bursa each at designated PID. ^#^Statistically significant differences between control and vIBDV- or cIBDV-infected groups (*p *< 0.05). *Statistically significant differences between vIBDV- and cIBDV-infected groups (*p *< 0.05).

The gene expression data of TLRs and their adaptor molecules are illustrated in Figure 7. We noted downregulation in the expression of TLR3 in vIBDV-infected bursa at all PIDs tested (Figure 7A). However, in cIBDV-infected bursa, TLR3 gene expression was significantly upregulated at PID 3 and 5 as compared to vIBDV, and it was downregulated at PID 7 (Figure 7A). The expression level of TLR 7 showed general trend of downregulation during the early stage of infection in chickens infected with either viral strain (Figure 7B).

Expression of TRIF was slightly upregulated in vIBDV-infected chickens as compared to controls whereas in cIBDV-infected chickens, TRIF expression was significantly higher than controls at 5 and 7 PIDs (Figure 7C). The gene expression of MyD88 in vIBDV-infected bursas was downregulated at all time points. MyD88 was upregulated at PID 3 and downregulated at PID 5 and 7 in cIBDV-infected chickens (Figure 7D).

## Discussion

Innate immune responses orchestrate the antiviral activities through the proliferation of different effector cells such as macrophages, T-cells and cytokines, chemokines and Toll like receptors. In this study, while comparing the pathogenesis of cIBDV and vIBDV, we noted that cIBDV produced more pronounced bursal damage as compared to vIBDV during early stages of the infection. Both viral strains induced pronounced infiltration of macrophages and T cells in the bursa of infected chickens. The expression of the proinflammatory cytokines and chemokines in cIBDV-infected bursa was significantly higher than vIBDV-infected bursa. Importantly, we observed that cIBDV infection upregulated the expression of TLR3 whereas vIBDV infection downregulated its expression.

Following infection, IBDV replicates extensively in B cells and causes bursal atrophy [15]. This study revealed that there were distinct differences in the early pathogenic events of the classical and variant strains of IBDV. Although both viruses caused bursal atrophy and lymphoid cell depletion, cIBDV replicated extensively in the bursa and induced more bursal lesions. Bursal lesions in cIBDV-infected bursa were accompanied by infiltration of inflammatory cells and well pronounced plical edema. In contrast, bursal lesions induced by vIBDV were accompanied by thickening of the intrafollicular septum and less marked inflammatory response. We also noted the inhibition of the expression of the antiviral cytokine IFN-α in cIBDV-infected chickens at PID 3. Although we did not examine the expression of IFN-α at earlier time points, in a previous study [43], inhibition of IFN-α in virulent IBDV-infected chickens was observed up to PID 4. The lack of IFN-α early in the infection would provide an opportunity for the virus to establish an infection.

Although antibody mediated immunity play an important role, it has been demonstrated that cell mediated immunity is also crucial against IBDV infection. This study compared for the first time the infiltration of T cells and macrophages in cIBDV- and vIBDV- infected bursa. The infiltration of T cells was significantly higher (*P *< 0.05) in cIBDV-infected bursa during early stage of infection. Previously, it was shown that a virulent strain of IBDV induced extensive infiltration of T cells as compared to an avirulent strain [44]. In our study, T cell infiltration was higher in cIBDV-infected chickens and cIBDV appeared to be more virulent (based on bursal lesions score). These findings indicate that infiltration of T cells may be related to the virulence of the virus strain. The T cells are hypothesized to mediate the virus clearance and may also be responsible for the exacerbated bursal lesions [45]. Higher infiltration of T cells in cIBDV-infected chickens may have contributed to the enhanced bursal damage observed during the early stage of the infection. Although not tested in this study, both classical and variant strains cause functional impairment of T cells such that T cells respond poorly to mitogens in vitro [33].

Macrophages are the central effector cells of the innate immune system. Cytokines produced by innate immune cells influence the nature of the adaptive immune response [46]. We observed the recruitment of macrophages in cIBDV- and vIBDV-infected bursa. Previously, we have shown that macrophages from IBDV-infected chickens produce proinflammatory cytokine [16]. In the present study, expression of the proinflammatory cytokine IL-6 and mediator iNOS was significantly upregulated in cIBDV-infected chickens as compared to vIBDV-infected chickens. The increased expression of IL-6 and iNOS correlated well with the previous reports where based on histological observations, it was shown that variant IBDV induced mild inflammatory response as compared to classical IBDV [5,33]. The mechanisms responsible for the recruitment of macrophages to the bursa of IBDV-infected chickens are not known. However, it is likely that the chemokines, IL-8 and MIP-α, may have a role in this regard. Chicken IL-8 acts as a chemoattractant for heterophils and monocytes [47]. In this study, we found that the expression of these chemokines was significantly upregulated in cIBDV-infected birds and slightly upregulated in vIBDV-infected birds. Although the number of macrophages in cIBDV- and vIBDV-infected bursa was the same, cytokine and chemokine data suggest that macrophages were likely to be more activated in cIBDV-infected chickens. Activated macrophages in chickens are known to be a source of pro-inflammatory cytokines [16,17].

The TLRs have been established to play a pivotal role in the activation of innate immunity by recognizing specific patterns of microbial components. In the present study, we demonstrate for the first time, the induction of TLRs and their adaptor proteins, TRIF and MyD88 in IBDV-infected chickens. TLR3 and TLR7 are the only TLRs implicated in antiviral responses in chickens [39]. Strikingly, TLR3 was downregulated in vIBDV-infected bursa whereas it was upregulated in cIBDV-infected bursa. In addition to TLR3, melanoma differentiation-associated gene 5 (MDA5) is also receptor for dsRNA [48]. The MDA5 activate IRF3 and NF-κB for the induction of innate immunity. Previously, we have shown that NF-κB regulates IBDV-induced cytokine production [21]. Therefore, it is possible that vIBDV induced activation of the innate response may be MDA5 mediated. Previously, TLR3 upregulation was shown in chickens infected with very virulent MDV and H5N1 avian influenza infection [49,50]. The expression of TLR3 adaptor protein TRIF was upregulated in cIBDV- and vIBDV-infected bursa. Further in vitro studies using a siRNA approach will be needed to delineate the role of TRIF-dependent or independent TLR3/MDA5-mediated innate immune activation by cIBDV and vIBDV.

While examining the expression of TLR7 in infected bursas, we found that TLR7 gene expression was downregulated in vIBDV-infected bursa. However, in cIBDV-infected bursas TLR7 gene expression was upregulated. TLR7 primarily act as receptor for ssRNA; however, dsRNA duplexes of 19-21 bp in length can activate mammalian TLR7 [51,52]. It is possible that the dsRNA genome of IBDV may activate avian TLR7. In our study, MyD88 was downregulated in cIBDV- and vIBDV-infected bursa. Previously, it was shown that dsRNA-triggered, TLR3-mediated signaling is independent of MyD88 [53].

In conclusion, this study reports striking differences in the pathogenesis and activation of host responses by cIBDV and vIBDV. Compared to vIBDV, cIBDV produced more pronounced bursal damage, accumulation of T cells, and inflammatory response (IL-6 and iNOS expression). Further studies are needed to identify the role of viral proteins responsible for mediating the differential host responses against classical and variant IBDV and the cellular source of cytokines and chemokines produced in the bursa. The findings of this study provide new insights that could be useful for the design of effective vaccines against classical and variant IBDV strains.

## Competing interests

The authors declare that they have no competing interests.

## Authors' contributions

Conceived and designed the experiments: MK YMS AR. Performed the experiments: AR MK MVM KJ. Analyzed the data: AR MK YMS. Wrote the paper: AR MK YMS. All authors read and approved the final manuscript.

## References

[B1] Lukert PD Saif YM Saif YM, Barnes HJ, Glisson JR, Fadly AM, McDougald LR, Swayne DE Infectious bursal disease Diseases of Poultry 2003 11 Ames: Iowa State University Press 161 179

[B2] Hirai K Funakoshi T Nakai T Shimakura S Sequential changes in the number of surface immunoglobulin-bearing B lymphocytes in infectious bursal disease virus-infected chickens Avian Dis 1981 25 484 496 10.2307/1589940 6266392

[B3] Rodenberg J Sharma JM Belzer SW Nordgren RM Naqi S Flow cytometric analysis of B cell and T cell subpopulations in specific-pathogen-free chickens infected with infectious bursal disease virus Avian Dis 1994 38 16 21 10.2307/1591831 8002886

[B4] Jackwood DJ Cookson KC Sommer-Wagner SE Le Galludec H de Wit JJ Molecular characteristics of infectious bursal disease viruses from asymptomatic broiler flocks in Europe Avian Dis 2006 50 532 536 10.1637/7528-032006R1.1 17274290

[B5] Ismail NM Saif YM Immunogenicity of infectious bursal disease viruses in chickens Avian Dis 1991 35 460 469 10.2307/1591208 1659364

[B6] Subler KA Mickael CS Jackwood DJ Infectious bursal disease virus-induced immunosuppression exacerbates Campylobacter jejuni colonization and shedding in chickens Avian Dis 2006 50 179 184 10.1637/7434-090705R.1 16863064

[B7] Bautista DA Elankumaran S Heckert RA Effect of a variant infectious bursal disease virus (E/Del) on Salmonella typhimurium infection in commercial broiler chickens Avian Dis 2004 48 361 369 10.1637/7130 15283423

[B8] Li G Lillehoj HS Lee KW Jang SI Marc P Gay CG Ritter GD Bautista DA Phillips K Neumann AP Rehberger TG Siragusa GR An outbreak of gangrenous dermatitis in commercial broiler chickens Avian Pathol 2010 39 247 253 10.1080/03079457.2010.487517 20706880

[B9] Fadly AM Winterfield RW Olander HJ Role of the bursa of Fabricius in the pathogenicity of inclusion body hepatitis and infectious bursal disease viruses Avian Dis 1976 20 467 477 10.2307/1589379 183647

[B10] Sharma JM Effect of infectious bursal disease virus on protection against Marek's disease by turkey herpesvirus vaccine Avian Dis 1984 28 629 640 10.2307/1590231 6091603

[B11] Pejkovski C Davelaar FG Kouwenhoven B Immunosuppressive effect of infectious bursal disease virus on vaccination against infectious bronchitis Avian Pathol 1979 8 95 106 10.1080/03079457908418330 18770430

[B12] Muller H Islam MR Raue R Research on infectious bursal disease--the past, the present and the future Vet Microbiol 2003 97 153 165 10.1016/j.vetmic.2003.08.005 14637046

[B13] Ramirez-Nieto G Shivaprasad HL Kim CH Lillehoj HS Song H Osorio IG Perez DR Adaptation of a mallard H5N2 low pathogenicity influenza virus in chickens with prior history of infection with infectious bursal disease virus Avian Dis 2010 54 513 521 10.1637/8902-042809-Reg.1 20521687

[B14] Kim IJ You SK Kim H Yeh HY Sharma JM Characteristics of bursal T lymphocytes induced by infectious bursal disease virus J Virol 2000 74 8884 8892 10.1128/JVI.74.19.8884-8892.2000 10982331 PMC102083

[B15] Sharma JM Kim IJ Rautenschlein S Yeh HY Infectious bursal disease virus of chickens: pathogenesis and immunosuppression Dev Comp Immunol 2000 24 223 235 10.1016/S0145-305X(99)00074-9 10717289

[B16] Khatri M Palmquist JM Cha RM Sharma JM Infection and activation of bursal macrophages by virulent infectious bursal disease virus Virus Res 2005 113 44 50 10.1016/j.virusres.2005.04.014 15893401

[B17] Palmquist JM Khatri M Cha RM Goddeeris BM Walcheck B Sharma JM In vivo activation of chicken macrophages by infectious bursal disease virus Viral Immunol 2006 19 305 315 10.1089/vim.2006.19.305 16817773

[B18] Kim IJ Karaca K Pertile TL Erickson SA Sharma JM Enhanced expression of cytokine genes in spleen macrophages during acute infection with infectious bursal disease virus in chickens Vet Immunol Immunopathol 1998 61 331 341 10.1016/S0165-2427(97)00135-9 9613445

[B19] Glass WG Rosenberg HF Murphy PM Chemokine regulation of inflammation during acute viral infection Curr Opin Allergy Clin Immunol 2003 3 467 473 10.1097/00130832-200312000-00008 14612671

[B20] Heitmeier MR Scarim AL Corbett JA Double-stranded RNA-induced inducible nitric-oxide synthase expression and interleukin-1 release by murine macrophages requires NF-kappaB activation J Biol Chem 1998 273 15301 15307 10.1074/jbc.273.24.15301 9614147

[B21] Khatri M Sharma JM Infectious bursal disease virus infection induces macrophage activation via p38 MAPK and NF-kappaB pathways Virus Res 2006 118 70 77 10.1016/j.virusres.2005.11.015 16388870

[B22] Sang Y Ross CR Rowland RR Blecha F Toll-like receptor 3 activation decreases porcine arterivirus infection Viral Immunol 2008 21 303 313 10.1089/vim.2008.0042 18788939

[B23] Kawai T Akira S Innate immune recognition of viral infection Nat Immunol 2006 7 131 137 16424890 10.1038/ni1303

[B24] Werts C Girardin SE Philpott DJ TIR, CARD and PYRIN: three domains for an antimicrobial triad Cell Death Differ 2006 13 798 815 10.1038/sj.cdd.4401890 16528382

[B25] Kawai T Akira S The roles of TLRs, RLRs and NLRs in pathogen recognition Int Immunol 2009 21 317 337 10.1093/intimm/dxp017 19246554 PMC2721684

[B26] Takeuchi O Akira S Innate immunity to virus infection Immunol Rev 2009 227 75 86 10.1111/j.1600-065X.2008.00737.x 19120477 PMC5489343

[B27] Takeda K Akira S TLR signaling pathways Semin Immunol 2004 16 3 9 10.1016/j.smim.2003.10.003 14751757

[B28] Stoute ST Jackwood DJ Sommer-Wagner SE Cooper GL Anderson ML Woolcock PR Bickford AA Senties-Cue CG Charlton BR The diagnosis of very virulent infectious bursal disease in California pullets Avian Dis 2009 53 321 326 10.1637/8684-030909-Case.1 19630244

[B29] Berg TP Meulemans G Acute infectious bursal disease in poultry: protection afforded by maternally derived antibodies and interference with live vaccination Avian Pathol 1991 20 409 421 10.1080/03079459108418779 18680037

[B30] Snyder DB Changes in the field status of infectious bursal disease virus Avian Pathol 1990 19 419 423 10.1080/03079459008418695 18679953

[B31] Okoye JO Shoyinka SV Newcastle disease in a vaccinated flock which had experienced subclinical infectious bursal disease Trop Anim Health Prod 1983 15 221 225 10.1007/BF02242062 6316596

[B32] Ismail NM Saif YM Wigle WL Havenstein GB Jackson C Infectious bursal disease virus variant from commercial Leghorn pullets Avian Dis 1990 34 141 145 10.2307/1591345 2157389

[B33] Sharma JM Dohms JE Metz AL Comparative pathogenesis of serotype 1 and variant serotype 1 isolates of infectious bursal disease virus and their effect on humoral and cellular immune competence of specific-pathogen-free chickens Avian Dis 1989 33 112 124 10.2307/1591076 2539070

[B34] Abdel-Alim GA Saif YM Detection and persistence of infectious bursal disease virus in specific-pathogen-free and commercial broiler chickens Avian Dis 2001 45 646 654 10.2307/1592906 11569738

[B35] Abdel-Alim GA Saif YM Immunogenicity and antigenicity of very virulent strains of infectious bursal disease viruses Avian Dis 2001 45 92 101 10.2307/1593016 11332505

[B36] Kim IJ Gagic M Sharma JM Recovery of antibody-producing ability and lymphocyte repopulation of bursal follicles in chickens exposed to infectious bursal disease virus Avian Dis 1999 43 401 413 10.2307/1592637 10494408

[B37] Rautenschlein S von Samson-Himmelstjerna G Haase C A comparison of immune responses to infection with virulent infectious bursal disease virus (IBDV) between specific-pathogen-free chickens infected at 12 and 28 days of age Vet Immunol Immunopathol 2007 115 251 260 10.1016/j.vetimm.2006.11.002 17157923

[B38] Rauf A Khatri M Murgia MV Saif YM Expression of perforin-granzyme pathway genes in the bursa of infectious bursal disease virus-infected chickens Dev Comp Immunol 2011 35 620 627 10.1016/j.dci.2011.01.007 21241730

[B39] Hghihghi HR Read LR Mohammadi H Pei Y Ursprung C Nagy E Behboudi S Haeryfar SM Sharif S Characterization of host responses against a recombinant fowlpox virus-vectored vaccine expressing the hemagglutinin antigen of an avian influenza virus Clin Vaccine Immunol 2010 17 454 463 10.1128/CVI.00487-09 20071494 PMC2837961

[B40] Sijben JW Klasing KC Schrama JW Parmentier HK van der Poel JJ Savelkoul HF Kaiser P Early in vivo cytokine genes expression in chickens after challenge with Salmonella typhimurium lipopolysaccharide and modulation by dietary n--3 polyunsaturated fatty acids Dev Comp Immunol 2003 27 611 619 10.1016/S0145-305X(03)00031-4 12697317

[B41] Jarosinski KW Yunis R O'Connell PH Markowski-Grimsrud CJ Schat KA Influence of genetic resistance of the chicken and virulence of Marek's disease virus (MDV) on nitric oxide responses after MDV infection Avian Dis 2002 46 636 649 10.1637/0005-2086(2002)046[0636:IOGROT]2.0.CO;2 12243528

[B42] Giulietti A Overbergh L Valckx D Decallonne B Bouillon R Mathieu C An overview of real-time quantitative PCR: applications to quantify cytokine gene expression Methods 2001 25 386 401 10.1006/meth.2001.1261 11846608

[B43] Eldaghayes I Rothwell L Williams A Withers D Balu S Davison F Kaiser P Infectious bursal disease virus: strains that differ in virulence differentially modulate the innate immune response to infection in the chicken bursa Viral Immunol 2006 19 83 91 10.1089/vim.2006.19.83 16553553

[B44] Rautenschlein S Yeh HY Sharma JM Comparative immunopathogenesis of mild, intermediate, and virulent strains of classic infectious bursal disease virus Avian Dis 2003 47 66 78 10.1637/0005-2086(2003)047[0066:CIOMIA]2.0.CO;2 12713160

[B45] Rautenschlein S Yeh HY Njenga MK Sharma JM Role of intrabursal T cells in infectious bursal disease virus (IBDV) infection: T cells promote viral clearance but delay follicular recovery Arch Virol 2002 147 285 304 10.1007/s705-002-8320-2 11890524

[B46] Mogensen TH Melchjorsen J Malmgaard L Casola A Paludan SR Suppression of proinflammatory cytokine expression by herpes simplex virus type 1 J Virol 2004 78 5883 5890 10.1128/JVI.78.11.5883-5890.2004 15140986 PMC415838

[B47] Barker KA Hampe A Stoeckle MY Hanafusa H Transformation-associated cytokine 9E3/CEF4 is chemotactic for chicken peripheral blood mononuclear cells J Virol 1993 67 3528 3533 8388511 10.1128/jvi.67.6.3528-3533.1993 PMC237699

[B48] Andrejeva J Childs KS Young DF Carlos TS Stock N Goodbourn S Randall RE The V proteins of paramyxoviruses bind the IFN-inducible RNA helicase, mda-5, and inhibit its activation of the IFN-beta promoter Proc Natl Acad Sci USA 2004 101 17264 17269 10.1073/pnas.0407639101 15563593 PMC535396

[B49] Karpala AJ Lowenthal JW Bean AG Activation of the TLR3 pathway regulates IFNbeta production in chickens Dev Comp Immunol 2008 32 435 444 10.1016/j.dci.2007.08.004 17870162

[B50] Abdul-Careem MF Haq K Shanmuganathan S Read LR Schat KA Heidari M Sharif S Induction of innate host responses in the lungs of chickens following infection with a very virulent strain of Marek's disease virus Virology 2009 393 250 257 10.1016/j.virol.2009.08.001 19733379

[B51] Heil F Hemmi H Hochrein H Ampenberger F Kirschning C Akira S Lipford G Wagner H Bauer S Species-specific recognition of single-stranded RNA via toll-like receptor 7 and 8 Science 2004 303 1526 1529 10.1126/science.1093620 14976262

[B52] Ward JR Bingle L Judge HM Brown SB Storey RF Whyte MK Dower SK Buttle DJ Sabroe I Agonists of toll-like receptor (TLR)2 and TLR4 are unable to modulate platelet activation by adenosine diphosphate and platelet activating factor Thromb Haemost 2005 94 831 838 16270639

[B53] Jiang Z Mak TW Sen G Li X Toll-like receptor 3-mediated activation of NF-kappaB and IRF3 diverges at Toll-IL-1 receptor domain-containing adapter inducing IFN-beta Proc Natl Acad Sci USA 2004 101 3533 3538 10.1073/pnas.0308496101 14982987 PMC373497

